# Modified Exposure and Response Prevention to Treat the Repetitive Behaviors of a Child with Autism: A Case Report

**DOI:** 10.1155/2011/241095

**Published:** 2011-07-03

**Authors:** Brian A. Boyd, Cooper R. Woodard, James W. Bodfish

**Affiliations:** ^1^Division of Occupational Science and Occupational Therapy, The University of North Carolina at Chapel Hill, 2050 Bondurant Hall, Campus Box 7122, Chapel Hill, NC 27599-7120, USA; ^2^Clinical Services and Training, The Groden Center, 86 Mount Hope Avenue, Providence, RI 02906, USA; ^3^Carolina Institute for Developmental Disabilities, Campus Box 7255, Chapel Hill, NC 27599, USA; ^4^Department of Psychiatry, The University of North Carolina at Chapel Hill, Chapel Hill, NC 27514, USA

## Abstract

We report the case study of a school-aged child with autism whose repetitive behaviors were treated with a modified version of a technique routinely used in cognitive behavior therapy (i.e., exposure response prevention) to treat obsessive-compulsive disorder. A trained behavioral therapist administered the modified ERP treatment over the course of an intensive two-week treatment period with two therapy sessions occurring daily. The treatment was successful at decreasing the amount of child distress and cooccurring problem behavior displayed; however, the child's interest in the repetitive behavior eliciting stimulus (i.e., puzzles) remained. The case study demonstrates specific ways that exposure response prevention strategies can be adapted to the unique kinds of repetitive behaviors that present clinically in autism. A larger clinical trial is needed to substantiate these findings.

## 1. Introduction

The term “repetitive behavior” is an umbrella term used to refer to a broad and often disparate class of behaviors linked by *repetition, rigidity, topographical similarity,* and *inappropriateness *(Boyd et al. [1]). In autism, these include stereotyped movements, repetitive manipulation of objects, self-injurious behaviors, specific object attachments, compulsions, rituals and routines, an “anxiously obsessive desire for sameness” (Kanner [2]), repetitive use of language, and circumscribed interests. These restricted and repetitive behaviors (RRBs) can cause significant functional impairment for individuals with autism and their families, because they often interfere with the individual's ability to function in daily life. Repetitive behaviors can consume the majority of an individual's waking hours, interfere with daily family activities, and affected individuals may become anxious, agitated, or disruptive if such behaviors are interrupted. Moreover, previous work has suggested that the more time a child spends engaging in repetitive behaviors, the less time he or she spends exploring the environment (Pierce and Courchesne [3] and Sasson et al. [4]). Thus, RRBs may replace or prevent normal exploration and experience-dependent learning during critical periods of development.

Given the functional impairment associated with repetitive behaviors in autism, the need for efficacious forms of intervention is apparent. While specific forms of pharmacologic treatment have been found to effectively diminish the occurrence of repetitive behaviors in autism (Lewis et al. [5] and McDougle et al. [6]), efficacy and safety of these medications in children is less established. Cognitive-behavioral treatment may be a viable alternative based on the similarity of repetitive behaviors in persons with autism and persons with obsessive-compulsive disorder (OCD). Previous studies on the relation of repetitive behaviors found in autism to those seen in OCD have demonstrated both phenomenological similarities and differences (Bejerot [7] and Bodfish et al. [8]). In some cases, OCD can occur as a comorbid condition superimposed on autism. However, in the majority of cases, classic symptoms of OCD (e.g., washing and checking) do not occur and instead the repetitive behaviors typical of autism (e.g., stereotyped movements, insistence on sameness, and unusual or intense interests) are associated with functional impairment. Still on purely phenomenological grounds, both OCD and autism involve both behavioral manifestations (e.g., compulsions in OCD; rituals/routines in autism) and cognitive manifestations (e.g., obsessions in OCD; insistence on sameness and preoccupations in autism).

With these similarities in mind, considerable evidence exists to support the efficacy of a specific technique used as part of a broader cognitive-behavior intervention—“exposure and response prevention” (ERP)—for the treatment of repetitive thoughts and behaviors in OCD (Abramowitz et al. [9]; Storch and Merlo [10]). ERP is routinely used in the clinical treatment of both children and adults with OCD. The *exposure* component of ERP typically involves the repeated, gradual exposure of the client to environmental stimuli associated with symptoms of anxiety and the subsequent expression of compulsive behaviors (Rapoport and Inoff-Germain [11]; Storch [12]). The *response prevention *component involves the active blocking or avoidance of the compulsive act. ERP itself is based on the behavioral extinction paradigm that anxiety attenuates after repeated exposure to the anxiety-/distress-producing stimulus and repeated prevention of compulsive behavior associated with that stimulus (March et al. [13]; Piacentini and Langley [14]). Some behavioral therapies also have included a competing response component as part of the treatment package (e.g., Habit Reversal Training), whereby the individual is taught an appropriate behavior to serve as a replacement for the treated repetitive behavior to prevent its reoccurrence (see Woods and Miltenberger [15]). 

In two previously reported case studies of an ERP-based intervention for children with autism (Lehmkuhl et al. [16]; Reaven and Hepburn [17]), the intervention was used to treat comorbid OCD symptoms (e.g., contamination thoughts or hand-washing rituals). To date, no studies have addressed the modification of ERP to treat more autism-typical forms of repetitive behavior. The purpose of this paper is to present a case report in which ERP was used to treat the repetitive behavior (a core deficit area) of a school-aged child with autism and without comorbid OCD.

## 2. Case Presentation

### 2.1. Overview of Screening and Treatment Procedures

This case study describes implementation of ERP with a school-aged child with autism, who will be referred to by the pseudonym Joey. Joey is a Caucasian boy who lives with his mother in a medium-sized city located in the northeastern USA and was 14 years old at the time of treatment. He was diagnosed with autism at age two by his neurologist. For study purposes, Joey's diagnosis was confirmed by a licensed clinical psychologist using current DSM-IV criteria, and he met autism criteria on the *Social Communication Questionnaire* (SCQ score of 23; Rutter et al. [18]), an autism screening tool. The ERP treatment occurred at the school for children with developmental disabilities and behavioral disorders he attends; Joey was referred to the day and residential school in September of 2000. At the time of the study (i.e., three years ago), Joey had only attended this school and was one of six students with autism in his classroom. He exhibited no spoken language but effectively used a “talk-box” (an augmentative communication device). Joey's cognitive functioning was found to be at the 1-to-2-year level for language skills and near the 3-year level for visual motor skills as measured by the *Mullen Scales of Early Learning* (Mullen [19]), which indicates a comorbid intellectual disability. His behavioral challenges included tantrums and self-injurious behavior (SIB). These behaviors were typically exhibited when one of Joey's ritualized activities was interrupted. Medications at the time of his involvement in the present study included Singulair, Citalopram (an SSRI often used to treat OCD symptoms), Melatonin, and Senna. Despite the administration of Citalopram, Joey continued to exhibit repetitive behavior. He had been on this medication for more than one year at the time the present research intervention began and remained on a stable dose of the SSRI medication throughout ERP treatment. 

A screening process was used to determine Joey's eligibility based on his clinical expression of repetitive behaviors. His repetitive behaviors were assessed using a psychometrically validated informant rating scale, the *Repetitive Behavior Scale-Revised* (RBS-R; Bodfish et al. [20]; Bodfish et al. [21]; Lam and Aman [22]) and subsequently the psychometrically validated structured clinical interview, the *Interview for Repetitive Behaviors *(IRB; Bodfish [23]; Turner-Brown et al. [24]), to identify specific repetitive behaviors that were associated with functional impairment and therefore could serve as targets for ERP therapy. In conjunction with the classroom teacher, the therapist assessed Joey's repetitive behaviors using the RBS-R, and results indicated strong tendencies to arrange and order materials, ritualized play activities, and distress when these activities were interrupted. The teacher completed the IRB, which indicated Joey's most frequent, intense, and interfering behaviors included needing to put objects away in the same location, needing to complete an activity, and playing with a restricted range of items during leisure time. Based on the results of the RBS-R and IRB, a specific repetitive behavior-inducing stimulus (puzzles) was selected for treatment. Puzzles were selected because Joey consistently became distressed when this activity was interrupted, and allowing Joey to complete puzzles took excessive amounts of time. It also was an activity that could be easily recreated in a clinic setting. Once deemed eligible for the study, Joey's primary caregiver was sent a letter to describe the study procedures and to obtain informed consent.

### 2.2. ERP Treatment Implementation

The treatment phase of the study lasted for a period of two weeks. Treatment sessions were conducted in a therapy room in the school that was separate from the child's classroom. A trained behavioral therapist conducted all treatment sessions. Each session was designed to last approximately 15–20 minutes with at least two sessions occurring per day. The clinic room contained a puzzle that Joey had previously been exposed to in his classroom and a familiar set of number identification cards that were identified as an appropriate competing response for Joey. These materials were on a table in the room with two chairs, and a small video monitor was suspended from the ceiling. The therapist had an earpiece that played a recording to indicate 16 randomly generated, variable intervals (30 seconds, 1 minute, and 1.5 minutes) of alternating repetitive behavior periods (puzzles) with mastered academic/alternative task periods (number identification cards). During the session, the therapist was animated and provided social reinforcement during the academic task trials but was quiet and disinterested during the trials where repetitive behavior was allowed. 

This modified alternating, discrete trial format of ERP was used to provide Joey with instruction on stopping rituals/restricted interests on a predetermined schedule, and switching from rituals/restricted interests to an adaptive, nonritual activity. During ERP sessions Joey received 8–10 discrete trials of the following therapy sequence (in order): (a) allow ritual/restricted behavior (on variable interval schedule that averaged 1 min), (b) interrupt access to ritual/block restricted behavior (using no more than a light physical redirection or touch, which was used as needed across all sessions), while leaving the eliciting repetitive behavior item (i.e., puzzles) within his physical reach, (c) administer academic task using graduated guidance and differential reinforcement for task engagement behavior (on a variable interval schedule that averaged 1 min), and (d) interrupt academic task and allow access to the repetitive behavior. At the end of each treatment session the therapist rated the intensity of Joey's expressed repetitive and problem behaviors using a standardized rating form based on the IRB; in addition, the teacher was asked to complete weekly ratings of the intensity of these behaviors exhibited outside of the clinic. However, the classroom teacher remained blind as to the purpose of the clinic sessions and the type of treatment being provided over the course of the two weeks.

### 2.3. Behavioral Outcome Data

**Figure 1:**
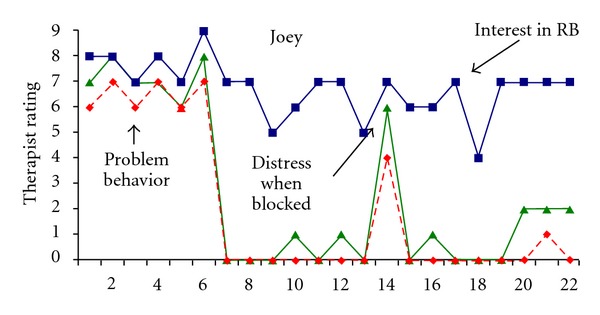
Therapist ratings of Joey's interest in the repetitive behavior (RB) item (i.e., puzzles), distress when access to puzzles was blocked, and intensity of problem behavior.

The graphed session-by-session data presented in Figure 1 are based on the therapist's daily ratings at the end of each treatment session. The data show that Joey's distress level and cooccurring problem behavior significantly reduced over the course of the ERP sessions; however, his general interest in puzzles did not decrease as evidenced by his continued desire to engage in this task when allowed to do so. Across the 22 ERP treatment sessions, dramatic differences were found for distress and problem behavior when the first 3 sessions (sessions 1–3) were compared to the last 3 sessions (sessions 20–22). Mean ratings for distress decreased from 7.3 (range = 7-8) to 2.0 (range = 2) and for problem behavior from 6.3 (range = 6-7) to 0.3 (range = 0-1). However, interest in puzzles remained relatively stable over time ((from M = 7.7 (range = 7-8) to M = 7.0 (range = 7)).

These data were confirmed based on videotaped coding by two independent raters who were naïve to study hypotheses. The primary rater coded the first two and the last two treatment sessions to provide a pre-post type comparison. Reliability between the two raters was greater than 80% and calculated in Observer 5.0, a behavioral coding software program that allows for real-time coding and analysis of frequency- and duration-based behaviors (Noldus [25]).

Based on these data, the percentage of time Joey engaged in the academic task during the treatment sessions increased from 37% to 66%, the amount of time that elapsed during the session before Joey began to engage in repetitive behavior (in the presence of puzzles) increased from 0.0 sec to 30.5 sec., and his rate (frequency/minute) of engagement in problem behavior decreased from 1.01/minute to 0.06/minute.

### 2.4. Relapse Prevention and Classroom Generalization

Following clinic-based treatment, the behavioral therapist trained the classroom teacher in the ERP treatment procedures. The teacher implemented the ERP treatment in the classroom twice per day for two weeks and at the end of each of the two weeks completed the teacher ratings. At the close of week one, the teacher rated the intensity of distress when puzzle completion was blocked at the “moderate” level and at the close of week two at the “moderate to mild” level.

## 3. Conclusion

We have developed a modified intervention protocol based on exposure and response prevention (ERP). This case study provides one example of the successful use of this modified ERP technique to treat the repetitive behavior of a school-aged child with autism. The current application of ERP differs from prior studies in two fundamental ways, potentially demonstrating a broader applicability of the current treatment approach. First, previous researchers have used ERP to treat comorbid OCD symptoms in autism (Lehmkuhl et al. [16]; Reaven and Hepburn [17]), whereas our form of ERP was used to treat more autism-typical repetitive behaviors. Second, in contrast to prior work, this case report successfully used ERP with a child with autism who had a comorbid intellectual disability.

Future research efforts should involve controlled trials of children with autism to examine the efficacy of this approach. If proven efficacious at treating repetitive behaviors in autism, then ERP may serve as an alternative or augmentative form of treatment for children with autism with clinically significant repetitive behaviors. 

In addition, we know that RRBs in autism first present during a critical period of development (as early as 12 months by some accounts) (Ozonoff et al. [26]), and the early emergence of these behaviors has been linked to poorer developmental outcomes (Morgan et al. [27]). Some form of ERP may show promise as an early, intensive behavioral intervention to reduce interfering behaviors that may impede the development of important adaptive behaviors and skills during this critical period. Further, failure to respond to ERP treatment may prove useful in helping to provide a clinical rationale to use medications to treat repetitive behaviors in autism, in particular for children, where there is less evidence of the success of pharmacological treatments.

## References

[1] Boyd BA, McBee M, Holtzclaw T, Baranek GT, Bodfish JW (2009). Relationships among repetitive behaviors, sensory features, and executive functions in high functioning autism. *Research in Autism Spectrum Disorders*.

[2] Kanner L (1943). Autistic disturbances of affective contact. *Nervous Child*.

[3] Pierce K, Courchesne E (2001). Evidence for a cerebellar role in reduced exploration and stereotyped behavior in autism. *Biological Psychiatry*.

[4] Sasson NJ, Turner-Brown LM, Holtzclaw TN, Lam KS, Bodfish JW (2008). Children with autism demonstrate circumscribed attention during passive viewing of complex social and nonsocial picture arrays. *Autism Research*.

[5] Lewis MH, Bodfish JW, Powell SB, Golden RN (1995). Clomipramine treatment for stereotypy and related repetitive movement disorders associated with mental retardation: a double blind comparison with placebo. *American Journal on Mental Retardation*.

[6] McDougle CJ, Naylor ST, Cohen DJ, Volkmar FR, Heninger GR, Price LH (1996). A double-blind, placebo-controlled study of fluvoxamine in adults with autistic disorder. *Archives of General Psychiatry*.

[7] Bejerot S (2007). An autistic dimension: a proposed subtype of obsessive-compulsive disorder. *Autism*.

[8] Bodfish JW, Crawford TW, Powell SB, Parker DE, Golden RN, Lewis MH (1995). Compulsions in adults with mental retardation: prevalence, phenomenology, and comorbidity with stereotypy and self-injury. *American Journal on Mental Retardation*.

[9] Abramowitz JS, Foa EB, Franklin ME (2003). Exposure and ritual prevention for obsessive-compulsive disorder: effects of intensive versus twice-weekly sessions. *Journal of Consulting and Clinical Psychology*.

[10] Storch EA, Merlo LJ (2006). Treatment of the patient with obsessive-compulsive disorder. *Journal of Family Practice*.

[11] Rapoport JL, Inoff-Germain G (2000). Practitioner review: treatment of obsessive-compulsive disorder in children and adolescents. *Journal of Child Psychology and Psychiatry and Allied Disciplines*.

[12] Storch EA (2005). Pediatric obsessive-compulsive disorder: guide to effective and complete treatment. *Contemporary Pediatrics*.

[13] March JS, Franklin M, Nelson A, Foa E (2001). Cognitive-behavioral psychotherapy for pediatric obsessive-compulsive disorder. *Journal of Clinical Child and Adolescent Psychology*.

[14] Piacentini J, Langley AK (2004). Cognitive-behavioral therapy for children who have obsessive-compulsive disorder. *Journal of Clinical Psychology*.

[15] Woods DW, Miltenberger RG (1995). Habit reversal: a review of applications and variations. *Journal of Behavior Therapy and Experimental Psychiatry*.

[16] Lehmkuhl HD, Storch EA, Bodfish JW, Geffken GR (2008). Brief report: exposure and response prevention for obsessive compulsive disorder in a 12-year-old with autism. *Journal of Autism and Developmental Disorders*.

[17] Reaven J, Hepburn S (2003). Cognitive-behavioral treatment of obsessive-compulsive disorder in a child with Asperger syndrome: a case report. *Autism*.

[18] Rutter M, Bailey A, Lord C (2003). *Social Communication Questionnaire (SCQ)*.

[19] Mullen EM (1995). *Mullen Scales of Early Learning*.

[20] Bodfish JW, Symons F, Lewis MH (1999). *The Repetitive Behavior Scales (RBS)*.

[21] Bodfish JW, Symons FJ, Parker DE, Lewis MH (2000). Varieties of repetitive behavior in autism: comparisons to mental retardation. *Journal of Autism and Developmental Disorders*.

[22] Lam KSL, Aman MG (2007). The repetitive behavior scale-revised: independent validation in individuals with autism spectrum disorders. *Journal of Autism and Developmental Disorders*.

[23] Bodfish JW (2003). *Inventory for Repetitive Behaviors (IRB)*.

[24] Turner-Brown LM, Lam KSL, Holtzclaw TN (2011). Phenomenology and measurement of circumscribed interests in autism spectrum disorders. *Autism*.

[25] Noldus (1991). *The Observer*.

[26] Ozonoff S, Macari S, Young GS, Goldring S, Thompson M, Rogers SJ (2008). Atypical object exploration at 12 months of age is associated with autism in a prospective sample. *Autism*.

[27] Morgan L, Wetherby AM, Barber A (2008). Repetitive and stereotyped movements in children with autism spectrum disorders late in the second year of life. *Journal of Child Psychology and Psychiatry and Allied Disciplines*.

